# Clinical translation of tissue-engineered oesophageal grafts: are patients ready for us?

**DOI:** 10.1007/s00383-024-05866-y

**Published:** 2024-11-06

**Authors:** N. Durkin, M. Pellegrini, V. Karaluka, G. Slater, D. Leyden, S. Eaton, Paolo De Coppi

**Affiliations:** 1 Stem Cell and Regenerative Medicine Section, Developmental Biology and Cancer Research and Teaching Department, Zayed Centre for Research Into Rare Disease in Children, UCL Great Ormond Street Institute of Child Health, London, UK; 2 NIHR GOSH Biomedical Research Centre, Great Ormond Street Hospital, London, UK; 3 Tracheo-Oesophageal Fistula Support (TOFS) Charity, Nottingham, UK; 4 Great Ormond Street Hospital for Children, Specialist Neonatal and Paediatric Surgery, London, UK; 5 Research Area of Fetal, Neonatal, and Cardiological Sciences, Bambino Gesù Children Hospital, IRCCS, Rome, Italy

**Keywords:** Oesophageal atresia, Tissue engineering, Patient and public involvement

## Abstract

**Purpose:**

We sought to engage with expert patient/carers to understand attitudes towards use of tissue engineering (TE) for long-gap oesophageal atresia (OA).

**Methods:**

An in-person engagement event for 70 patients/parents was held by the OA patient group, TOFS. Attitudes towards TE were assessed before and after a talk on use of TE oesophagi in a pre-clinical OA model. Perceptions were assessed using a 5-point Likert scale (median [range]) and compared using Mann–Whitney test.

**Results:**

43 attendees responded; 56% parents/caregivers, 21% patients, 7% healthcare workers, 16% unreported. Most (85%) had some awareness of TE but for 15%, it was a new concept. Attendees were receptive to TE; 89% reported no concerns about growth of their/child(s) cells in a lab and 61% reported no concerns about using animal products. Perceptions of TE significantly improved after the presentation from 4 (2–5, *n* = 32) to 5 (3–5, *n* = 28) *p* < 0.0001, and 96% would like to be involved in focus groups on development of a TE product for use in OA.

**Conclusion:**

Input from key stakeholders is essential to introduction of TE constructs clinically. The overall response to TE constructs was positive, and informs development of an OA-specific focus group to guide translation.

## Introduction

Patient engagement is essential for development and successful translation of novel clinical therapies. Tissue engineering (TE) is a rapidly expanding clinical field which aims to replace or repair damaged or absent tissues with functional constructs by combining cells, scaffolds, and bioactive molecules. This field holds great promise for paediatric surgery, with the potential to revolutionise the treatment of congenital defects. One such example is long-gap oesophageal atresia (LGOA), where the use of a TE construct could provide an off-the-shelf, personalised, size-matched oesophageal substitute without the loss of function of an organ associated with traditional replacement techniques (e.g. stomach, colon, jejunum). Feasibility of a TE approach has recently been demonstrated in pre-clinical animal models of long-gap OA by several groups, including our own [1–4].

Tissue engineering is a clinical reality, with successful paediatric transplantation of engineered trachea in 2010, and the use of multiple other tissues described in both adult and paediatric settings including skin, gingival cartilage, veins, cornea, urethra, and bladder [5, 6]. Despite this, little is published about patient perspectives on tissue engineering as a concept and its use in clinical practise. As such, public perceptions remain largely unknown. This is particularly relevant in children who are unable to give informed consent and where there are wider family implications. To that end, we sought to understand expert patient/carer attitudes towards the potential use of TE oesophageal grafts for long-gap oesophageal atresia (OA) to gauge acceptability of concepts, barriers to translation and guide development of patient-acceptable success and risk criteria to inform the development of a future first-in-human trial.

## Methods

An open access, all day, in-person seminar and engagement event was held by the UK OA patient support group, Tracheo-Oesophageal Fistula Support (TOFS), in November 2023 focussed on “Improving care in OA/TOF”. Various seminars were given on current research within the OA sphere by affiliated healthcare professionals; a specific, patient-focussed talk on the background, development, and use of TE oesophageal grafts in a pre-clinical, porcine model of LGOA was delivered, written with the aid of the local NIHR lead for Patient and Public Engagement (PPIE). Pre-existing knowledge and attitudes towards TE and surrounding concepts were assessed among key stakeholders (patients > 11 years, parents, and healthcare professionals) by a live Mentimeter link during the talk. Data displayed as percentages represent the percentage of responders for that question. Contingency tables were analysed using Fisher’s exact test. Perceptions of TE were assessed pre- and post-delivery of the seminar with free-text words clouds and on a 5-point Likert scale, displayed as median (range) and compared using the Mann–Whitney test. A p < 0.05 is considered significant.

## Results

Of the 70 attendees, 43 engaged in the survey using smartphones (61%). Of these, 56% were parents/caregivers (*n* = 25), 21% patients (child/adult, *n* = 8), 7% healthcare professionals (*n* = 3) and 16% did not specify their role (*n* = 7). Interestingly, there was a high overall awareness of TE; 46% reported being broadly aware of the term and 39% had previously read about it. Only 15% reported that it was an entirely new concept. Parents (90%) were more likely to be aware of TE than patients (66%) although this difference was not significant (*p* = 0.218). Awareness of TE was primarily through the TOFS charity affiliation (46%), although 25% reported exposure to the concept through the media (news, television, social) and medical professionals (21%).

**Fig. 1:**
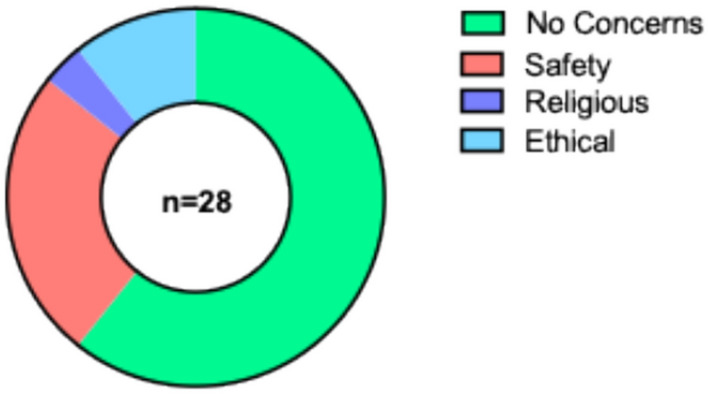
Attitudes about the use of animal tissue in tissue-engineered grafts

**Fig. 2:**
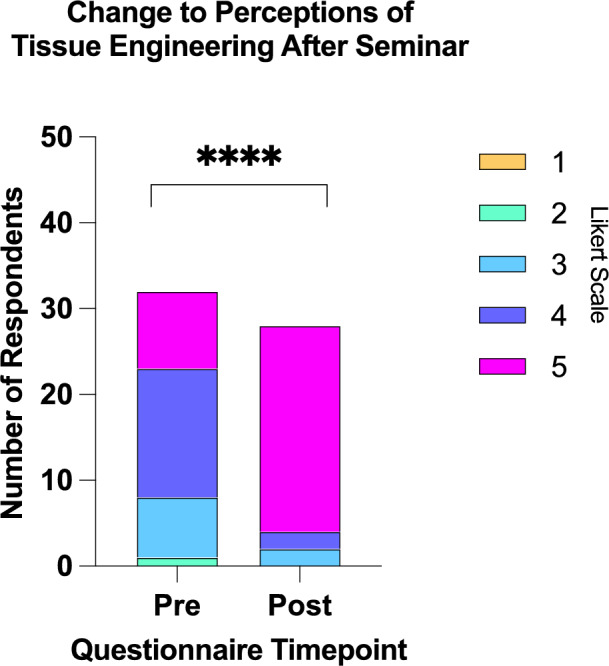
Change to perceptions of tissue engineering pre- and post-seminar: Perceptions of TE significantly improved after the seminar on TE oesophageal grafts on a Likert scale from 4 (2–5, *n* = 32) to 5 (3–5, *n* = 28) *p* < 0.0001

**Fig. 3:**
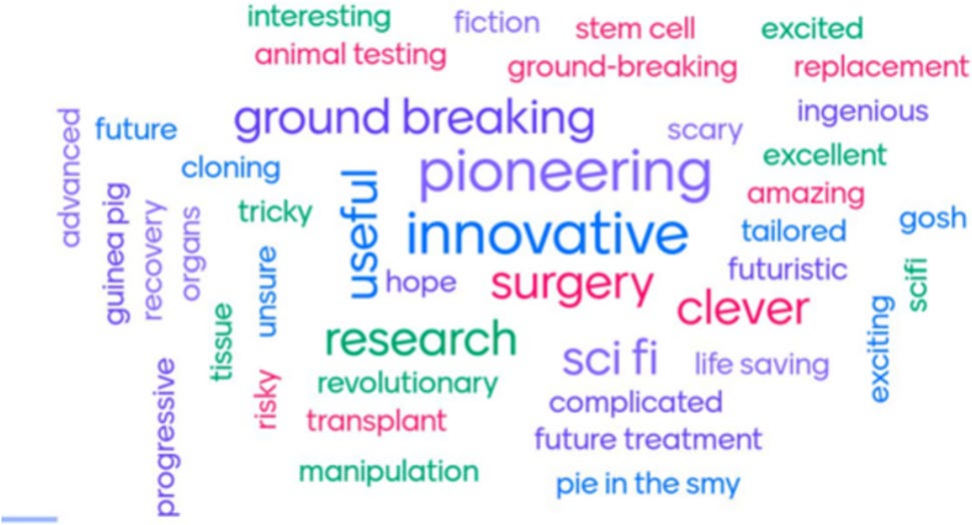
Word cloud of perceptions of tissue engineering pre-seminar

**Fig. 4:**
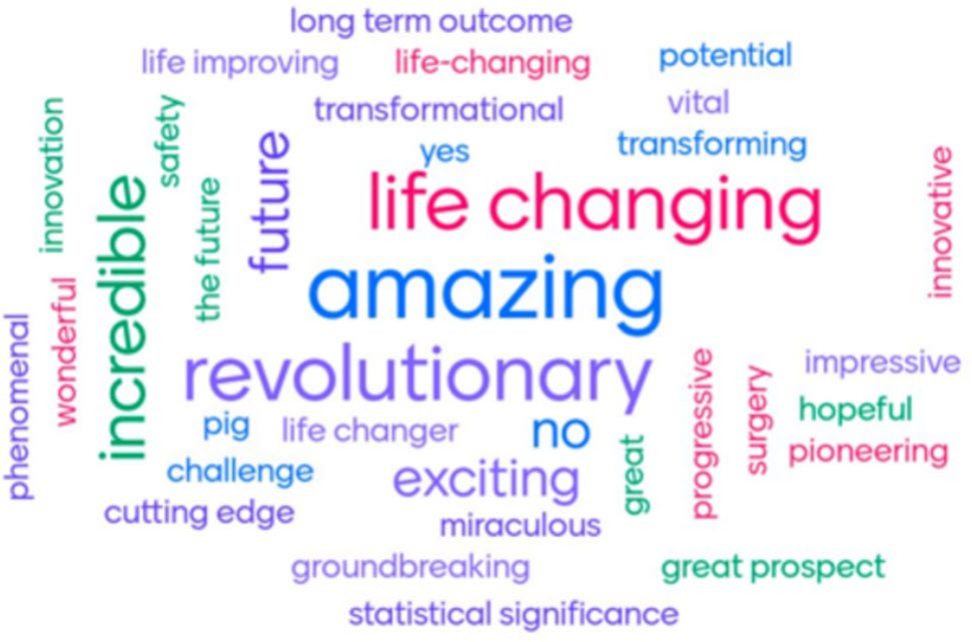
Word cloud of perceptions of tissue engineering post-seminar

Patients and parents were receptive to TE approaches; 89% reported they would not be concerned about the growth of their/child(s) cells in a lab, whilst 11% were unsure. The majority of responders (61%) reported no concerns about the use of animal products in TE grafts, with 39% reporting some concern for safety (*n* = 7), ethical (*n* = 3) and religious (*n* = 1) reasons (Fig. 1). Perceptions of TE significantly improved after the seminar on TE oesophageal grafts on a Likert scale from 4 (2–5, *n* = 32) to 5 (3–5, *n* = 28) *p* < 0.0001 (Fig. 2). The focus of free text words also changed pre- and post-seminar; whilst some words with positive connotations were listed pre-talk (‘*pioneering*,’ ‘*clever*,’ ‘*innovative*’), there were also several with negative connotations including ‘*fiction*’, ‘*guineapig*’, ‘*scary*,’ ‘*sci-fi*’, ‘*risky*’ and ‘*manipulation*’ (Fig. 3). Conversely, post-talk, the word cloud was overwhelmingly positive, with words including ‘*amazing* (*n* = 5),’ *life-changing*,’ ‘*phenomenal*,’ ‘*ground-breaking*,’ and ‘*revolutionary*’ (Fig. 4). When asked about concerns not addressed within the scope of the talk, pertinent questions arose including surgical considerations (e.g. the incidence of double stricture with two anastomoses), long-term outcomes (e.g. how the graft would grow over time), future considerations (e.g. the impact TE graft failure might have on the need for second-line oesophageal replacement techniques), and logistical considerations (e.g. trial eligibility and access). Crucially, post-presentation, 96% reported they would like to be involved in the establishment of focus groups on development of a TE product for use in long-gap OA.

## Discussion

Although clinical use is increasing, patient and public perspectives towards TE have not been well reported in the literature, despite being key to identifying and understanding their acceptability to patients. Reporting on the degree of public awareness of TE is also limited with one German study reporting only 8% being aware of the concept, albeit outdated (reported in Zoller [7]). Public knowledge of stem cell technology, a sister branch of regenerative medicine, appears to be more widespread, with 49% of UK respondents reporting to be familiar or very familiar with the concept during the same timeframe (reported in Zoller [7]). In our study, a higher proportion of patients and parents (85%) than expected reported at least some prior knowledge of TE, likely in part due to the audience and forum within which the presentation was given. Whilst this may have made the patient group more receptive and engaged than the wider OA patient base or indeed the public, perceptions of TE were still predominantly positive. This may be partially explained by extensive and predominantly positive media coverage of the translation of emerging biomedical technologies in recent years. However, several articles have highlighted the overly optimistic reporting of regenerative medicine therapies in press releases, exaggerating success with little context of risk, limitations or timeframes of the application, with one study assessing the positive slant in reporting to be as high as 59% [8, 9]. In some countries (e.g. the United States), public interest is so high that the formation of Regenerative Medicine Clinics has been initiated, advising patients on regenerative options for their conditions, with over a third of consultations including discussions of interventions that have not been rigorously tested for efficacy, or even safety [10]. A Japanese group recently studied the expectations of regenerative medicine in a survey of over 3000 members of the public and the scientific community; they found expectations of the scope of regenerative medicine were exaggerated in the public group, with significantly higher proportions expecting the possibility of life span extension and organ replacement in the next 10 years [11]. Clearly, therefore, there is a gulf between public expectation and the reality of tissue engineering, a phenomenon also described in stem cell therapies, gene therapies and organoid research [12–15]. This risks the creation of unrealistic social expectations resulting in public distrust and disappointment in the scientific community. In conjunction with this, the recent high profile media coverage of the misuse of tracheal TE constructs will have had a huge impact on public perceptions and confidence in TE [16, 17]. Although not specifically reported, in 2017, the Karolinska Hospital dropped from 4 to 15th in a Swedish opinion poll of the best hospitals in the country, likely reflecting this breach of public trust. Interestingly, there was no evidence that this negatively influenced the perception of TE in our group, and there were no questions or comments from the audience about this. To mitigate this, transparent and conscientious reporting of TE research in the media is essential and must be led from the scientific community with the help of constant dialogue with the patient population for whom the product is intended. Thus, it is beholden on both scientists, clinicians and journalists to report TE in a realistic and accurate manner.

Patient engagement forums can gauge perspectives and barriers to translation and can also inform design of first-in-human clinical trials. In recent years, there are increasing national blueprints promoting the uptake of impactful ethical, legal and social implication research, training and outreach in the regenerative medicine space using engagement with the public (e.g. Japan and Canada) [11, 18]. Although the outputs are as yet unknown, one case study of public participation in TE development has previously been described: The EU-Gene Activated Matrices for Bone and Cartilage Regeneration on Arthritis (GAMBA) Group [7]. This involved the development of lay panels with both patients and interested public. After receiving brochures with written information, they received presentations on the approach, risks, ethical aspects of gene therapies prior to questions and breakout groups. The vast majority of participants found it a useful endeavour and felt listened to. We, similarly, saw a positive response after our engagement event. Despite discussion of scientifically complex and ethically sensitive concepts such as decellularisation of tissue, muscle biopsy for primary cell derivation and the need for in vivo animal work, patient perspectives about the potential for TE to treat congenital diseases, specifically OA, improved significantly after the talk, both qualitatively and quantitatively. In addition, the insightful and perceptive questions from patients and parents about TE constructs and reported outcomes validates that concepts were understood. We believe that working together with a PPIE engagement partner was instrumental in the design and delivery of a seminar in which these concepts became accessible and understandable to patients of differing ages and backgrounds.

Interestingly in the GAMBA work, they found differences in focus and areas of importance between the patient panels and lay citizens; patients focussed more on ethical aspects such as informed consent, data protection, the use of animals and effects on personal risks rather than wider societal risks related to the environment and third parties raised by the lay public. Similarly in the Japanese study mentioned above, the public had a higher interest in logistics including cost of care, counter measures for risks, clarification of responsibility and responsiveness compared to researchers who highlighted the desire to know more about the mechanism of regeneration and clinical testing outcomes [11]. Interestingly in our study, the barriers to future translation and patient acceptability (e.g. cell expansion, use of xenografts) assumed by the research team did not appear to be of concern, however unanticipated questions about outcomes from the in vivo work not previously measured (e.g. degree of growth of grafts) and accessibility to a future trial were raised. This highlights that the priorities of patients are different than those performing the research, and it is therefore key that patients who are eligible to be in receipt of this technology have an opportunity to be intimately involved in shaping what this may look like early in study design.

The willingness of the cohort to become involved in a future OA-specific focus group was extremely encouraging, as this is essential for the development of first-in-human studies. As opposed to Phase I trials in healthy volunteers, regenerative medicine trials often have added complexities of the difficulty in informed consent due to vast uncertainties, appropriate patient selection (advanced vs. primary disease) and understanding risk/benefit balance, difficult to accurately state by definition in a first-in-human scenario [19]. First, a focus group provides a platform for collaboration between the investigating team and key stakeholders; this promotes transparency and encourages discussion about whether pre-clinical research findings are sufficiently robust and the risk/benefit ratio acceptable to patients to move forwards to clinical trials. Second, a heterogenous group of adult and paediatric OA patients and care givers can help inform the most appropriate patient selection (e.g. re-do vs. primary LGOA repair). The focus group can also help guide the development of patient/caregiver facing materials, ensuring appropriate wording for information sheets and consent forms for a fully informed consent process. Finally, by identifying patient-centred outcomes and metrics of importance to the patients and their families, focus groups can outline pre-defined acceptable investigation and follow-up protocols and help interrogate and define the meaning of success of the trial [20]. Introduction of surgical, or indeed any clinical innovation can be difficult to implement appropriately, as highlighted by issues with previous tissue-engineered products. Early and continued engagement with Patient Advocacy and Support organisations can provide useful information to the regulatory authorities so that they can be assured of acceptability of the proposed innovation to the target population. Whilst this study is specific to TE, paediatric surgeons should consider adopting this approach for the introduction of other surgical innovations.

Limitations to our work include a bias of the expert patient in understanding pre-existing knowledge of TE, particularly as this work has previously been endorsed by TOFS and potential exclusion of engagement from those at the extremes of age due to limited access or accessibility to the live Mentimeter platform.

In conclusion, we have shown that the overall response to the concepts, processes and potential use of TE constructs was broadly positive, and acts as a baseline for development of an OA-specific focus group to help guide next steps towards translation. We believe this study is relevant to the implementation of regenerative medicine product to children and the consideration that families’ representatives should be involved from the early stage of product’s development.

## Data Availability

Data obtainable from corresponding authors on reasonable request.
